# Any difference? Use of a CAM provider among cancer patients, coronary heart disease (CHD) patients and individuals with no cancer/CHD

**DOI:** 10.1186/1472-6882-12-1

**Published:** 2012-01-12

**Authors:** Agnete E Kristoffersen, Arne J Norheim, Vinjar M Fønnebø

**Affiliations:** 1 Department of Community Medicine, National research center in complementary and alternative medicine (NAFKAM), Tromsø, Norway

## Abstract

**Background:**

Although use of complementary and alternative medicine (CAM) among cancer patients has been described previously, prevalence of use has not commonly been compared to other disease groups in a true population sample where CAM use or cancer is not the main focus. The aims of the present study are to (1) examine how CAM use in cancer patients differs from people with a previous CHD diagnosis and people with no cancer or CHD diagnosis in an unselected general population and (2), investigate the use of a CAM provider among individuals with a previous cancer diagnosis.

**Methods:**

A total of 8040 men and women aged 29 to 87 in the city of Tromsø, Norway filled in a questionnaire developed specifically for the Tromsø V study with questions on life style and health issues. Visits to a CAM provider within the last 12 months and information on cancer, heart attack and angina pectoris (heart cramp) were among the questions. 1449 respondents were excluded from the analyses.

**Results:**

Among the 6591 analysed respondents 331 had a prior cancer diagnosis, of whom 7.9% reported to have seen a CAM provider within the last 12 months. This did not differ significantly from neither the CHD group (6.4%, p = 0.402) nor the no cancer/CHD group (9.5%, p = 0.325).

**Conclusion:**

According to this study, the proportion of cancer patients seeing a CAM provider was not statistically significantly different from patients with CHD or individuals without cancer or CHD.

## Background

Cancer patients' self-reported use of complementary and alternative medicine (CAM) is increasing, [1-3] although studies report substantial differences in the level of use, ranging from 7 [4] to 91% [5]. Younger, highly educated women are the most frequent users [6-8]. Frequent use is also reported among patients with symptoms and symptom progression related to their cancer [9-13].

CAM treatment is mostly offered outside the national health care service in Norway and paid out-of-pocket by the patients. Prior to 2004 only physicians and dentists could legally treat cancer patients [14]. The proportion of cancer patients reporting CAM use in Norway varies between 11.1 and 72% [15,16] depending on how CAM is defined [15]. When defined as "at least one visit to a CAM provider during the previous 12 months" the variation narrows down to 16.1% [16] to 22.7% [15].

CAM use among cancer patients has rarely been reported in an unselected general population sample, and even more rarely been compared to use among other patient groups in this type of sample [17].

Coronary heart disease (CHD) and cancer constituted 58% of all deaths in Norway in 2009 [18], and are the two most common causes of death. In planning, administering and monitoring health care provisions, knowledge about the choices and health care-related behaviours made by these patient groups is important, particularly the choices and behaviours related to treatments outside the national health care service.

The magnitude of use of conventional health care in CHD patients is well known. Few studies have, however, examined CAM use in these patients, most of them in highly selected population subgroups. Substantial differences in the proportion of users ranging from 12%-85% [19-21] have been reported.

As with patients with other chronic diseases, CHD patients are likely to use CAM to manage their condition, increase their quality of life, and prevent recurrence of disease [22,23]. So far, there are no comparable data regarding use of CAM among Norwegian CHD patients.

This wide range of reported CAM use in both cancer and CHD patients may be due to several factors; differences in the definition of a CAM user [15,24,25], whether CAM is used for general health purposes or for illness-specific reasons [20,23], the time frame of reported use [26] and differing legislation [27] regulating CAM provisions and funding. The differences might also be due to lack of population-based data on CAM use in these two patient groups.

The aims of the present study are therefore to (1) examine how CAM use in cancer patients differs from people with a previous CHD diagnosis and people with no cancer or CHD diagnosis in an unselected general population and (2), investigate the use of a CAM provider among individuals with a previous cancer diagnosis.

## Methods

The Tromsø Study series (I-VI) are prospective studies in the municipality of Tromsø, Northern Norway. The design includes repeated population health surveys to which total birth cohorts and random samples are invited. This paper is based on data from the Tromsø V study conducted in 2002.

A total of 10353 men and women were invited to participate in this study. This included individuals participating in the extended fourth survey in 1994-1995 (Tromsø IV) [28]. In addition, all inhabitants who turned 30, 40, 45, 60 or 75 during 2001 were invited to participate. As 2313 did not attend, the study included 8040 subjects, 4565 women and 3475 men, aged between 29 and 87 (response rate 77.6%).

The Tromsø studies have been linked electronically to the Cancer Registry of Norway (CRN) enabling the identification of cancer patients by two methods; through self-reporting of cancer in the survey and through registration in the CRN. Registration of cancer has been mandatory by law since 1952, and the registry is therefore considered virtually complete.

**Figure 1:**
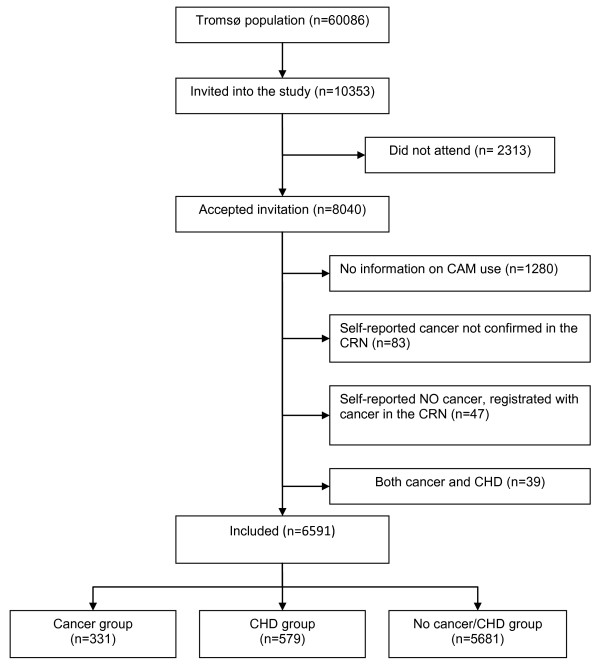
**Flow chart that shows the selection of the studied population**.

A total of 1280 participants had not answered the question regarding visits to a CAM provider and were therefore excluded from the current analysis. Further, 169 persons were excluded due to the following two reasons: They had experienced both cancer *and *CHD, or they had reported having cancer without this being registered in the CRN (Figure 1). The analysis of visits to a CAM provider in cancer and CHD patients thus included 6591 respondents.

The letter of invitation contained a questionnaire developed specifically for the Tromsø study. Individuals who attended the survey by undergoing a health screening and answering the first questionnaire received subsequently a *second *questionnaire that they were asked to complete and return by mail.

The two questionnaires included questions on general state of health, diseases suffered by the respondent or their family, muscle pain and physical discomfort, food habits, alcohol consumption, smoking habits, physical activity in leisure time, level of education, use of medicine and use of health services including a CAM provider [29].

The question regarding visits to a CAM provider was not directly related to any specific disease condition. The questions concerning CAM and CHD were included in the first questionnaire completed before the health screening, while the question concerning cancer was placed in the second questionnaire returned by mail after the health screening.

A CAM user in this study is defined as a respondent who checked one or more visits on the question: *How many visits have you made during the past year to an alternative medical provider*? A "no CAM user" is a respondent who checked for *no visits*. This question was one item in a list including 12 other non-CAM health care providers (for example general practitioner (GP), psychologist, psychiatrist, emergency room physician, home nurse, physiotherapist, chiropractor, dentist etc.).

In Norway, an alternative medical provider is commonly understood by the public as a practitioner providing CAM both as alternative and complementary treatment. A CAM provider offers therapies that are not commonly offered within the public health care service and are paid out-of-pocket by the patients themselves.

CAM use was compared between three groups:

1. The cancer group (n = 331)

2. The CHD group (n = 579)

3. The no cancer/CHD group (n = 5681)

The cancer group consisted of informants who had checked *Yes *for: *Have you ever had, or do you have cancer? *and were registered with a cancer diagnosis in the CRN. Informants were also included in this group if they had left the question unanswered (due to deliberate choice or failing to return the second questionnaire) but were registered with a cancer diagnosis in the CRN. Informants in this group were also required to have checked *No *or have a missing value for both: *Do you have, or have you had a heart attack *AND *Do you have, or have you had angina pectoris (heart cramp)*? The members of this group are referred to as "cancer patients" even though the time of their clinical cancer disease may have been several years ago and/or they considered themselves to be healed from their cancer.

The CHD group consisted of respondents who had checked *Yes *for: *Do you have, or have you had a heart attack *OR *Do you have, or have you had angina pectoris (heart cramp)*? and who were not included in the cancer group.

The no cancer/CHD group consisted of respondents who had checked *No *or had a missing value for: *Have you ever had, or do you have cancer?*, and were *not *registered with cancer in the CRN nor were included in the CHD group.

The primary endpoint in this study was reported visits to a CAM provider over the previous 12 months in the cancer group compared to the CHD group and the no cancer/CHD group. The secondary endpoint was visits to a CAM provider over the previous 12 months within the cancer group.

With a statistical power of 80% and using an alpha of 0.05 we were able to report as statistically significant differences in reported use of approximately 6.5 percentage points between the two smallest groups.

The endpoints were analyzed using chi-square tests and logistic regression in SPSS Windows (version 17.0, SPSS Inc., Chicago, IL). When the compared groups differed significantly from each other in terms of baseline characteristics with possible influence on CAM use, the comparison between groups are also reported with adjusted p-values.

The data inspectorate has been notified about the study and the regional ethics committee has recommended it.

## Results

### Basic characteristics of the studied participants

The cancer group consisted mainly of women, the CHD group mainly of men, while the no cancer/CHD group was gender-balanced. Individuals in the no cancer/CHD group were higher educated than the cancer group and the CHD group. The no cancer/CHD group had the best self-reported health, the CHD group the poorest (Table 1).

**Table 1:** Basic characteristics of studied participants

	Cancer (n = 331)	CHD (n = 579)	No cancer/CHD (n = 5681)
Mean age, years (range)	66.6 (30-84)	68.9 (39-85)	57.12 (29-87)
Median age	67	69.5	60
Percentage of women	60.1%	36.1	55.0%
Years of education (mean)	9.9	9.1	11.2
Self-reported poor health	47.1	61.6%	32.8%
Living with a spouse/partner	68.1%	68.9%	73.2%

Mean time from first diagnosis was 9.6 years (median = 6.6), ranging from 0 to 41 years in the cancer group, and 9.6 years (median = 8) in the CHD group ranging from 0 to 54.

### Use of a CAM provider in the cancer group compared to the CHD group

**Figure 2:**
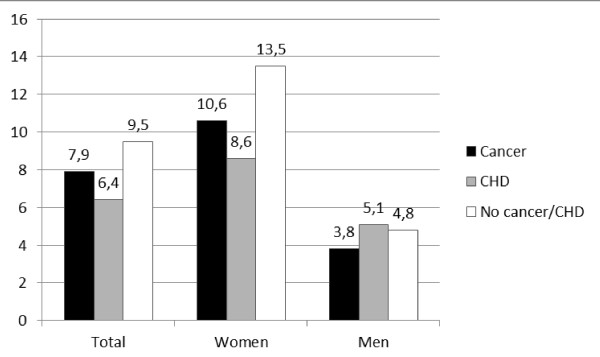
**Use of a CAM provider within the last 12 months**. A comparison between the cancer group, the CHD group and the no cancer/CHD group.

26 participants (7.9%) in the cancer group and 37 participants (6.4%) in the CHD group had visited a CAM provider within the last 12 months (p = 0.402, Figure 2).

The insignificant difference between the two groups remains when adjusted for gender, age, self-reported health and education.

### Use of a CAM provider in the cancer group compared to the no cancer/CHD group

593 participants (9.5%) in the no cancer/CHD group and 26 participants in the cancer group (7.9%) had seen a CAM provider the last 12 months (p = 0.325, Figure 2). 16 participants (4.8%) in the cancer group and 270 (4.3%) in the no cancer/CHD group had seen their provider more than three times (p = 0.209).

### Use of a CAM provider within the cancer group

Among the 26 patients (7.9%) in the cancer group that had visited a CAM provider in the previous 12 months, 10 patients had seen their provider one to three times while 16 patients had visited a provider more than three times.

A higher proportion of women compared to men tended to have visited a CAM provider, 21 women (10.6%) versus five men (3.8%) (p = 0.025). Of these, both men and women were most likely to have visited a provider more than three times.

Nine patients (11.5%) with metastases and 15 patients (7.5%) with no metastases at first diagnosis had visited a CAM provider in the previous 12 months (p = 0.287). Eight patients (10.8%) with metastases and eight patients without metastases (3.7%) had visited a CAM provider more than three times.

16 patients (8.3%) with at least five years since first diagnosis were just as likely to have visited a CAM provider as patients with one to five years since last diagnosis (9 patients, 8.3%). Only one person with less than one year since last diagnosis had visited a CAM provider within the last 12 months (2.9%).

## Discussion

This study shows no significant difference in visits to a CAM provider between population-based patients with a prior cancer or CHD diagnosis, and also no statistically significant difference in visits to a CAM provider between patients with a prior cancer diagnosis and individuals without cancer or CHD when adjusted for possible confounding factors. The findings can be seen as contra intuitive, but are therefore possibly even more important.

### Bias considerations

The high response rate in this study ensures a representative sample of the population. There was a mismatch between self-reported cancer and the registrations in the CRN regarding 130 participants. They had either identified themselves as having had cancer without a confirmed diagnosis in the CRN (n = 83), or identified themselves as having had no cancer with a confirmed diagnosis in the CRN (n = 47). Possible reasons for this may be (1) that the diagnosis had been uncertain and therefore not confirmed in the CRN, (2) the respondent had ticked off incorrectly in the questionnaire, (3) did not remember their diagnosis as cancer or (4) forgot about their previous cancer while filling in the questionnaire.

The exclusion of patients denying actively a cancer diagnosis despite a CRN registration can be seen as controversial. None of these reported using CAM. If we had included them in the cancer group, the proportion using CAM in this group would therefore have been slightly lower, while the differences would remain statistically insignificant. It might also be controversial to include patients with missing values on the CHD variable in the cancer group when excluding patients with both cancer and CHD. The number of cancer patients with a missing value on the CHD variables was seven, and none of these reported to be CAM users. Excluding them in the cancer group would only minimally have changed our estimates, and none of the differences would reach statistical significance.

The questionnaire asked for the number of visits to a CAM provider without *defining *a CAM provider. This could constitute an over- or underreporting of visits depending on how each participant defined a CAM provider. However, since the question regarding visits to a CAM provider was listed among a number of other health care providers, the separation between a CAM provider and a conventional health care provider should have been clear. There is no study in Norway on how the public defines a CAM provider, but the most commonly used CAM providers are massage therapists, acupuncturists, reflexologists, spiritual healers and homeopaths [30]. A chiropractor is in Norway licensed by the government as a regulated profession within conventional health care, and is not seen or classified as a CAM provider. There is no study in Norway on how the public defines a CAM provider, but this possible misclassification is not likely to be differential. The 12-month recall period concerning use of a CAM provider might also result in inaccuracies with regard to number of visits.

The onset of cancer or CHD might have occurred several years ago and the patient might therefore have given an inaccurate answer concerning whether or not they have had the disease.

The potential misclassifications in this study are likely to be non-differential and the results from this sample are therefore a conservative estimate of any population differences between groups.

The possible information bias generated when participants are fully aware of the purpose of the study (CAM use in cancer and CHD) was low in this study as this was the purpose of this paper but not in any way the main purpose of the Tromsø V study.

### Other studies

#### CAM use in CHD populations

We have not succeeded in finding other studies reporting use of a "*CAM provider*" among patients with CHD and are therefore unable to present a direct comparison. However, a British study reporting use of "any alternative or complementary therapies/medicines" reported findings similar to ours (9.2%) [21]. The similar and rather low CAM use in both studies might be due to the fact that patients in neither study were given a definition of CAM or a pre-prepared list of CAM treatments that might have added to the recall and produced a higher rate of CAM use [31]. The British study had a wider definition of CAM but was, on the other hand, administrated by a nurse in a hospital setting which might have made some patients reluctant to disclose CAM use.

#### CAM use in cancer patients

Comparison of our results with other studies in cancer was also difficult since the variation in time frame of use, purpose of use, time since diagnosis, definitions of a CAM provider and the population studied, strongly influence the results. We have therefore chosen to compare our study to a limited selection of other studies with focus on equality and comparability.

Breast cancer patients in England [32] and Canada [33] had visited a CAM provider more often than women with cancer in our study. This might be explained by the fact that women with breast cancer are generally more likely to be CAM users than patients with cancer at other sites [34]. Since our study consisted of all cancer sites, this might hamper the comparison. These differences might also be explained by the limitation of CAM use within the last 12 months in our study, while long-term use of CAM was included in the Canadian study.

A Norwegian study of CAM use in cancer patients with a poor survival prognosis at the time of first diagnosis, found that 22.7% had seen a CAM provider at least once after their diagnosis. They also found that the reported use increased to 40.6% when CAM techniques and over the counter (OTC) products were included [15]. The rather higher use in that study might be due to the longer time frame (since diagnosis) and the poor prognosis [35].

#### Comparative studies

Our results are supported by lack of significant differences in use of a CAM provider between different disease groups in a Canadian study. They compared CAM use in patients with inflammatory bowel disease (IBD), arthritis and a group with mixed chronic diseases where a minority were cancer patients [36]. The substantially higher self-reported use in the Canadian study (38.1%) might be due to the different nature of the diseases studied and the different availability of adequate curative and/or palliative treatment within the health care system. The fact that our study is a population-based and not a study limited to patients with the condition under study or to CAM use in general, might also explain some of the differences.

Similar use of a CAM provider in a cancer and a no cancer group was found in a US study conducted in 2002 [37]. Their study was like ours mainly based on long-term cancer survivors which might explain the similarities between the two groups. They found, however, that reported CAM use was higher in the cancer group when non-prayer CAM services, products, and practices were included.

It is not unlikely that the similar use of a CAM provider in the cancer group and the no cancer/CHD group in our study partly could be due to the strict legislation that regulated the CAM field at the time of the study; only physicians and dentists were allowed to treat cancer patients. It is therefore possible that the reported use was, at least partly, connected to other health problems than treating the cancer.

Cancer patients in our study visited a CAM provider less frequently than cancer patients in the USA [17]. Contrary to our results, the US-study found that cancer patients were more likely to use a CAM provider than the general population and individuals with chronic serious diseases, including CHD [17]. The higher use might be due to different definitions of a CAM provider and the legal restrictions on CAM treatment of cancer in Norway [38].

Cancer patients in our study also used a CAM provider less than Norwegian cancer patients in a similar study conducted in Nord-Trøndelag, Central Norway in 1995-1997 (HUNT) [16](8.2% versus 16.1%). They found, contrary to us, that cancer patients were more likely to have seen a CAM provider than the total population. Possible reasons for the higher use might be that the availability of CAM providers is higher in their area. And possibly more important, in the HUNT study they listed several commonly used CAM providers as a reminder for the patients in the questionnaire. This might have improved the recall and made it easier to understand what the researchers were asking for [31,39]. They also included chiropractors in their definition of a CAM provider which is specifically excluded in our study as they are licensed health care personnel in Norway; if visits to a chiropractor were included in our analyses, the use of a CAM provider would increase to 10.9% in the cancer group.

The rather low use of a CAM provider in all groups compared to studies from other countries shows how important it is to do domestic, locally-based studies. The observed low use might be due to cost differences in Norway. While most treatments offered within the public health care service are free of charge, most CAM treatments are paid out-of pocket.

### Interpretation

This is the first population-based study that to our knowledge reports use of a CAM provider in CHD patients and is therefore a door-opener to the field. In research regarding use of CAM in cancer patients, it is important to make comparisons with other relevant chronic disease groups. The differences and similarities found might contribute to a better understanding of the needs of the different groups. In addition, our study can inspire further research in the field.

Knowledge of CAM use in different patient groups is important for the conventional medical community. It is therefore important that they ask their patients about CAM use as negative interactions between conventional and CAM treatments can occur. The number of CAM users are likely to be higher than what was found here if OTC products and self-help techniques were included in the study [26].

Our study contributes to the information needed for health care providers and politicians to make knowledge-based decisions concerning CAM use. Our results differ from those from other countries, supporting the importance of locally performed surveys. However, this possible interpretation must be drawn with caution, as worldwide experience and knowledge give a broader perspective for creating guidelines and political priorities.

Studies like ours contribute to a broader knowledge base regarding cancer patients' attitudes to, and experience with, use of a CAM provider. This is needed to balance the impressions from random magazine reports and/or prejudiced points of view obtained from strong believers *or *opponents of CAM. The assumed widespread use of CAM among cancer patients is not documented in our results.

## Conclusions

The proportion of cancer patients in the Tromsø V study that visited a CAM provider was not statistically significantly different from patients with CHD or individuals without cancer or CHD. These findings are in accordance with some studies and contrary to others. Most other studies report a higher use of a CAM provider than we found in our study.

This study indicates that locally based contextual surveys are necessary to make scientific and political decisions from a knowledge-based point of view.

## Abbreviations

CAM: Complementary and alternative medicine; CHD: Coronary heart disease; CRN: Cancer registry of Norway; I-CAM-Q: International questionnaire to measure use of complementary and alternative medicine; NAFKAM: National research center in complementary and alternative medicine; OTC: Over the counter.

## Competing interests

The authors declare that they have no competing interests.

## Authors' contributions

AEK conceived the study, performed the initial and final analyses and drafted the manuscript. AJN helped draft the manuscript and reviewed subsequent versions. VF conceived the study together with AEK, helped draft the manuscript, and reviewed subsequent versions.

All authors read and approved the final manuscript.

## Pre-publication history

The pre-publication history for this paper can be accessed here:

http://www.biomedcentral.com/1472-6882/12/1/prepub
